# 2’-Hydroxychalcone Induces Autophagy and Apoptosis in Breast Cancer Cells via the Inhibition of the NF-κB Signaling Pathway: In Vitro and In Vivo Studies

**DOI:** 10.3390/nu16040514

**Published:** 2024-02-13

**Authors:** Xiao Wang, Yongjie Liang, Bohan Zhang, Lixin He, Wenxuan Li, Wenwen Zhang, Chengzong Li, Lihong Luo, Talha Umar, Huili Feng, Changwei Qiu

**Affiliations:** 1 Department of Clinical Veterinary Medicine, College of Veterinary Medicine, Huazhong Agricultural University, Wuhan 430070, China; 2 Department of Animal Physiology and Molecular Biology, College of Animal Husbandry Engineering, Henan Vocational College of Agriculture, Zhengzhou 451450, China

**Keywords:** hydroxychalcone, autophagy, apoptosis, NF-κB signaling, reactive oxygen species

## Abstract

2’-Hydroxychalcone is a hydroxyl derivative of chalcones, which are biosynthetic precursors of flavonoids and rich in the human diet. The anticancer activity of 2’-hydroxychalcone has been reported in several cancers but remains to be investigated in breast cancer. In the current study, 2’-hydroxychalcone showed significant cytotoxicity against breast cancer cell lines MCF-7 and CMT-1211. It could inhibit breast cancer cell proliferation, migration, and invasion in vitro and suppress tumor growth and metastasis in vivo. Mechanistic investigation revealed that the NF-κB pathway was significantly inhibited by 2’-hydroxychalcone treatment accompanied by an excessive intracellular accumulation of reactive oxygen species, induction of endoplasmic reticulum stress, and activation of JNK/MAPK. In addition, 2’-hydroxychalcone elevated the autophagic levels in breast cancer cells equipped with increasing numbers of autophagy vesicles and complete autophagic flux. Finally, autophagy-dependent apoptosis was observed in 2’-hydroxychalcone-induced cell death. In conclusion, 2’-hydroxychalcone enhances the autophagic levels and induces apoptosis in breast cancer cells, which could be contributed to the inhibition of the pro-survival NF-κB signaling, indicating a promising potential for 2’-hydroxychalcone in future anticancer drug development.

## 1. Introduction

Breast cancer is a leading cause of cancer-related deaths among women worldwide [1]. It not only severely impacts women’s health but also has been a longstanding focal point of global public health systems. Based on the most recent global cancer data published by the International Agency for Research on Cancer in 2020, breast cancer has now surpassed lung cancer to become the most prevalent form of cancer worldwide [2]. It is imperative to develop novel efficacious drugs against breast cancer.

Chalcones, characterized by the structure 1,3-diphenyl-2-propenone, are biosynthetic precursors of flavonoids, which are abundant in edible plants like fruits and vegetables [3]. Epidemiological research indicates that consuming a diet rich in flavonoid-containing edible plants is associated with a reduced risk of cancer in humans [4,5]. In recent years, there has been increasing interest in developing chalcone-based anticancer drugs because they are structurally simple, easily modified, and, most importantly, show multiple bioactivities, including antioxidant, anti-inflammatory, and anticancer activities [6,7]. The predominant forms of naturally occurring chalcones are primarily hydroxylated, and hydroxylated chalcones exhibit more potent anticancer activity than chalcones with other functional groups [8,9]. Studies reported that hydroxychalcones elicited potent cytotoxicity against various cancers [10,11,12]. However, few studies focus on the anti-breast-cancer effect and the underlying mechanism of 2’-hydroxychalcone, which represents only one hydroxyl substituent on the 2’ position of the A ring and was found to show strong inhibition effects on lung cancer [13], cervical cancer [14], and colon cancer [15] cells. Furthermore, chalcones exist in two geometric isomers: cis (Z) and trans (E). The latter, being more thermodynamically stable, represents the most prevalent structure among chalcones [16].

Previous studies revealed that chalcones exert anticancer activity through a variety of mechanisms. Chalcone, the core structure of chalcones, arrested the cell cycle in the G2/M phase and triggered mitochondrial apoptotic cell death in breast cancer [17] and bladder cancer [18]. Butein, a naturally occurring hydroxychalcone, triggered endoplasmic reticulum stress (ERS) in non-small-cell lung cancer through the PERK/eIF-2/CHOP pathway and caused apoptosis [19]. Synthetic 2’-hydroxychalones induced apoptosis and autophagy in A549 lung cancer cells [13]. Furthermore, hydroxychalcones were broadly reported to function as NF-κB inhibitors [20,21,22]. The NF-κB signaling pathway is a known survival pathway, and the transcriptional activation of its target genes connected with cell proliferation, metastasis, the inhibition of apoptosis, and antioxidant regulation seems to promote neoplastic progression and chemoresistance [23,24]. Thus, it could be a promising therapeutic strategy for breast cancer to inactivate the NF-κB pathway by hydroxychalcones. In the present study, our objective was to explore the anti-breast-cancer effect of (E)2’-hydroxychalcone (2’-HC) and the underlying mechanisms both in vitro and in vivo.

## 2. Materials and Methods

### 2.1. Chemicals and Antibodies

(E)2’-hydroxychalcone (2’-HC; purity ≥ 98%) was purchased from Tokyo Chemical Industry (Tokyo, Japan). Hydroxychloroquine (HCQ; purity ≥ 98%), 3-Methyladenine (3-MA; purity ≥ 98%), and N-acetylcysteine (NAC; purity ≥ 99%) were obtained from Sigma-Aldrich (St. Louis, MO, USA). Rapamycin (Rap; purity ≥ 98%) was purchased from Aladdin (Shanghai, China). 

The primary antibodies used were as follows: β-actin, Bax, Bcl-2, PARP, cleaved-PARP p25, caspase-9, caspase-3, LC3B, Beclin1, and p-IκB. They were purchased from ABclonal (Wuhan, China). p62/SQSTM1 was obtained from Proteintech (Wuhan, China). ERK, JNK, p-JNK, p38, p-p38, NF-κBp65, IκB, p-IκB, p-eIF2α, and MMP9 were obtained from Abmart (Shanghai, China). P-ERK, p-NF-κBp65, ATF-4, and CHOP were purchased from Wanleibio (Shenyang, China). HRP Goat Anti-Rabbit IgG (Abclonal), Alexa Flour 594-Goat Anti-Rabbit IgG (Abbox, Jiangsu, China), Cy3 Goat Anti-Rabbit IgG (H + L) (Abclonal), and FITC Goat Anti-Rabbit IgG (Servicebio, Wuhan, China) were used as secondary antibodies for a Western blot or immunofluorescence.

### 2.2. Cell Culture

The MCF-7 human breast cancer cell line was procured from the American Type Culture Collection (ATCC, Manassas, VA, USA). The CMT-1211 canine mammary cancer cell line was generously supplied by Professor Degui Lin from China Agricultural University (Beijing, China). Both cell lines were cultured in DMEM (Gibco, Billings, MA, USA) supplemented with 10% FBS (Hycezmbio, Wuhan, China) and 100 U/mL of penicillin–streptomycin (Gibco, Billings, MA, USA) at 37 °C in a humidified atmosphere of 5% CO_2_. No mycoplasma contamination was detected in the cells.

### 2.3. Cell Viability Assay

The cells were distributed in 96-well plates with a density of 1 × 10^4^ cells per well and cultured until they reached a confluence of 50–60%. To evaluate the cytotoxic impact of 2’-HC, cellular samples were subjected to varying concentrations of 2’-HC (10, 20, 30, 40, 50, 60, 70, 80, and 90 μM) for 24 h. A series of tests was undertaken at various time intervals (12, 24, 36, 48, 60, and 72 h) to assess the impact of 2’-HC on cell proliferation. For mechanistic experiments, cells were pre-treated with 3-MA (5 mM, 2 h), Rap (2 μM, 1 h), or NAC (10 mM, 2 h) and cultured with 2’-HC for an additional 24 h. Cell viability was assessed using the Cell Counting Kit-8 (CCK-8; Hycezmbio, Wuhan, China) following the manufacturer’s instructions. The absorbance at 450 nm was quantified using a multifunctional microplate reader (BMG LRBTECH, SPECTORstar, Offenburg, Germany) to quantify the results.

### 2.4. Wound Healing Assay

To quantify the migratory behavior of breast cancer cells, the cells were cultured in 6-well plates to produce monolayers and reached 80% confluence. Wounds were generated using a 200 μL pipette tip, which was employed to vertically scrape the cell layer in places where cells were densely packed. Subsequently, the cells were gently washed with sterile phosphate-buffered saline (PBS) twice to eliminate any detached cells. Following this, the cells were replenished with Opti-MEM I media (Gibco, Billings, MA, USA) that had been decreased in serum content, either with or without the addition of 2’-HC. Photographs of the wounds were taken at the time of the initial introduction of the scrape wound and again after a 24 h period using an inverted microscope (Olympus, CKX41, Tokyo, Japan). The distances of the optical wound were measured utilizing the ImageJ program.

### 2.5. Transwell Assay

To detect cell invasion ability, firstly, matrigel (Biozellen, Ord, NE, USA) was diluted 15-fold (MCF-7) and 18-fold (CMT-1211), respectively, before smoothly spreading on the upper chamber of a 24-well transwell insert (Biofil, Guangzhou, China). After the matrigel became solidified at 4 °C, cells that had been treated with 2’-HC for 24 h were digested from 6-well plates and suspended in 100 µL of a serum-reduced medium, finally being seeded into the matrigel-coated chambers at a density of 6 × 10^4^ cells per chamber. Meanwhile, a 500 μL complete medium was added to the lower chamber of the transwell inserts. After incubation for 24 h, the non-invaded cells on the upper side of the upper chamber were removed by scraping with a cotton swab, while the invaded cells on the lower side of the upper chamber were fixed with methanol and then stained with crystal violet. An optical microscope (Olympus, BX53, Tokyo, Japan) was utilized to capture images.

### 2.6. Detection of Acidic Vesicular Organelles

The LysoSensor^TM^ Green DND-189 (Yeasen, Shanghai, China) reagent was utilized to detect the acidity of intracellular vesicular organelles under the principle that green fluorescence intensity enhances when the acidity increases. Cells were treated with 2’-HC for 24 h, followed by incubation with a 1 μM LysoSensor reagent for 20 min at 37 °C in darkness. The fluorescence intensity was observed under an inverted fluorescence microscope (Olympus, IX73, Tokyo, Japan).

### 2.7. Autophagic Flux Analysis

To detect the autophagic flux of breast cancer cells, the mCherry-eGFP-LC3 plasmid, which was kindly provided by Professor Min Cui from Huazhong Agricultural University (Wuhan, China), was transfected into cells according to the protocol of the jetPRIME transfection reagent (Polyplus, Illkirch, France). Cells were seeded in 35 mm confocal dishes (Corning, NY, USA) at a density of 1 × 10^5^ cells per dish and cultured with 1 mL of a complete medium until 50% confluence was reached. Cells were subsequently transfected with 2 μg per dish of the mCherry-eGFP-LC3 plasmid for 20 h before being treated with 2’-HC (20 μM), Rap (1 μM), or HCQ (20 μM) for another 24 h. Another set of experiments was conducted at different times of cells being exposed to 20 μM of 2’-HC (4, 8, 12, and 24 h) to evaluate the dynamic changes in autophagic flux triggered by 2’-HC. After incubation, the fluorescence images were obtained using a laser scanning confocal microscope (Zeiss, LSM 800, Jena, Germany).

### 2.8. Flow Cytometric Analysis of Apoptosis

A flow cytometric analysis of apoptosis was performed using the Annexin V-Fluorescein isothiocyanate (FITC)/Propidium iodide (PI) Apoptosis Assay Kit (Hycezmbio, Wuhan, China). Briefly, cells were cultured in 6-well plates and treated with 2’-HC for 24 h after reaching a 60–70% confluence. Then, cells from different groups were harvested and counted to reach a density of 1 × 10^6^/mL after being resuspended in 250 μL of a binding buffer. Finally, cells were stained with Annexin V-FITC and PI according to the manufacturer’s instruction. In total, 5 μL of Annexin V-FITC and 100 μL of cell suspension were combined and subsequently incubated for 10 min at room temperature (20–25 °C) in the absence of light. In total, 10 μL of PI was added to the cell suspension 5 min prior to detection on the flow cytometer (Beckman Coulter, cytoflex-LX, Brea, CA, USA). A data analysis was performed with FlowJo10.4 software.

### 2.9. Measurement of Intracellular ROS

Intracellular reactive oxygen species (ROS) was assessed using the ROS Detection Kit (Hycezmbio, Wuhan, China) following the manufacturer’s instructions. Briefly, cells were cultured in 6-well plates, and upon reaching 60–70% confluence, they were subjected to treatment with 2’-HC for either 12 h or 24 h or pretreated with 10 mM NAC for 2 h, or their combination. Then, cells were harvested and counted to reach a density of 1 × 10^6^/mL after being resuspended in 250 uL of 1000× diluted fluorescent probe DCFH-DA. After 30 min of incubation at 37 °C, cells were rinsed three times with serum-free DMEM. Finally, cells were centrifuged and suspended with 1 mL of PBS before detection on a flow cytometer (Beckman Coulter, cytoflex-LX, Brea, CA, USA) at an excitation wavelength of 488 nm and an emission wavelength of 530 nm. Rosup, provided with the detection kit, was used in the ROS-positive control group at a concentration of 50 μL/mL for 20 min of stimulation.

### 2.10. Immunofluorescence Staining

Cells were seeded into 24-well plates at a density of 1 × 10^4^ cells per well and treated with 2’-HC for 24 h upon reaching a confluence of 50–60%. Following this, the cells underwent fixation with 4% paraformaldehyde for 20 min, permeabilization with 0.2% Triton X-100 for 10 min, and blocking with 5% BSA for 2 h, and were incubated with the primary antibody at 4 °C overnight. Alexa Flour 594-Goat Anti-Rabbit IgG was used as a fluorescent secondary antibody and incubated with cells for 2 h. Finally, nuclei were stained with DAPI (Beyotime, Shanghai, China) for 10 min. Additionally, tumor sections also underwent immunofluorescence staining. Sections were deparaffinized using xylene, gradually rehydrated with ethanol, immersed in a citric acid antigen retrieval buffer (pH 6.0), and subjected to antigen retrieval via microwave heating. Subsequent steps were analogous to those employed in cellular immunofluorescence staining. Lastly, the sections were mounted using an anti-fade fluorescent quenching agent. Image acquisition was performed using a fluorescence microscope (Olympus, IX73, Tokyo, Japan).

### 2.11. Western Blot Analysis

Cells were planted in 6-well plates and treated with 2’-HC for 24 h or pretreated with 3-MA for 2 h or their combination before being harvested with an ice-cold RIPA buffer. Total protein concentrations were measured with a bicinchoninic acid (BCA) protein quantification kit (Hycezmbio, Wuhan, China) to normalize the amount of total protein of samples to 15 μg. The proteins were subsequently separated using sodium dodecyl sulfate (SDS)–polyacrylamide gel electrophoresis and transferred onto polyvinylidene difluoride (PVDF) membranes. These membranes were then blocked in 5% skim milk for 2 h, followed by overnight incubation with the primary antibody at 4 °C and subsequent incubation with the secondary antibody at room temperature (20–25 °C) for 2 h. Finally, signals were detected with imager Fusion Solo S (Viber, Paris, France), and were analyzed using ImageJ1.53e.

### 2.12. Xenograft Mouse Model

This study was approved by the Institutional Ethical Committee for Animal Care and Use of Huazhong Agricultural University (permit number: HZAUMO-2023-0259). All the experimental procedures adhered to the United States National Institutes of Health guidelines. Five-week-old Balb/C female mice were purchased from the Experimental Animal Center of Huazhong Agricultural University (Wuhan, China) and maintained in standard conditions for one-week adaptation. Approximately 1 × 10^6^ CMT-1211 cells were harvested and suspended in 100 μL of PBS, then subcutaneously injected into the mouse’s fourth mammary pad. Next, mice were randomized into four groups, the control group (PBS), low-dose group (20 mg/kg), medium-dose group (40 mg/kg), and high-dose group (60 mg/kg), after the tumor volume reached about 100 mm^3^. Intraperitoneal injection and tumor volume and body weight measurement were performed every 2 days. Tumor volume was assessed using the following formula: V (mm^3^) = length × width^2^/2. Three weeks post-injection, all mice were euthanized, and the xenograft tumors were meticulously excised and weighed before being partly immersed with 4% paraformaldehyde and partly transferred to −80 °C for a subsequent analysis. The major organs were collected and immersed to perform hematoxylin and eosin (H&E) staining.

### 2.13. H&E Staining

The mice’s tumor tissues and essential organs (lung, liver, spleen, kidney, heart) were isolated and fixed with 4% paraformaldehyde as referred to in the previous part. Subsequently, the tissues were dehydrated, embedded in paraffin, and sectioned into slices with a thickness of 5 μm for H&E staining. Digital images were captured using an optical microscope (Olympus, BX53, Tokyo, Japan).

### 2.14. TUNEL Assay

The detection of apoptosis in the in vivo experiment was performed with a terminal deoxynucleotidyl transferase dUTP nick end labeling (TUNEL) Assay Kit (Roche, Basel, Switzerland) following the manufacturer’s instructions. Initially, the tumor tissues underwent standard deparaffinization and dehydration procedures. Subsequently, they were treated with proteinase K for 30 min at 37 °C, rinsed with PBS, and then incubated with TUNEL reagents containing terminal deoxynucleotidyl transferase (TdT) and fluorescent isothiocyanate-dUTP for 2 h at 37 °C. After endogenous peroxidase activity inhibition, sections were treated with converter-POD for 30 min, followed by diaminobenzidine (DAB) color development and counterstaining of nuclei. Finally, the sections were dehydrated and mounted using neutral gum.

### 2.15. RNA Sequencing Analysis and Prediction of the Potential Targets of 2’-HC

To investigate the underlying mechanisms of MCF-7 cells in response to treatment of 30 μM 2’-HC for 24 h, high-throughput RNA sequencing (RNA-Seq) was conducted by Personal Biotechnology Co., Ltd. (Shanghai, China) on a MiSeq 2000 sequencing platform (Illumina, CA, USA). The RNA sequencing data were uploaded to SRA with a submission number: SUB14148932. The DESeq R package was used to identify differentially expressed genes (DEGs) between control (*n* = 3) and 2’-HC-treated (*n* = 3) groups. Genes with |log_2_foldchange| > 1 and *p*-value < 0.05 were considered DEGs and were visualized in a volcano plot and heat map. A Gene Set Enrichment Analysis (GSEA) of signaling pathways from the Kyoto Encyclopedia of Genes and Genomes (KEGG) database was performed using the Java version of the software (gsea2–2.2.3.jar) with the molecular profile data abstained from RNA-Seq. A KEGG pathway enrichment analysis was performed using the KEGG Automatic Annotation Server (KAAS; http://www.genome.jp/tools/kaas, accessed on 13 November 2022) based on the bi-directional best hit (BBH) method. Gene Ontology (GO) annotations of 2’-HC-regulated genes were obtained through eggNOG-mapper software v2.1.5. The Search Tool for the Retrieval of Interacting Genes (STRING; http://string-db.org, accessed on 23 November 2022) database was utilized to investigate the underlying interactions among DEGs regulated by 2’-HC. DEGs with an interaction score ≥ 0.4 were further imported to Cystoscope 3.10.1 for an analysis and hub genes with a degree ≥ 5 were obtained. The protein–protein interaction (PPI) network among related hub genes was drawn using STRING.

SwissTargetPrediction (http://www.swisstargetprediction.ch/, accessed on 5 January 2023) was used to predict the putative compound targets of 2’-HC according to chemical similarities and pharmacophore models.

### 2.16. Statistical Analysis

Data are presented as the mean ± standard deviation (mean ± SD) of at least three independent experiments. A one-way analysis of variance (ANOVA) followed by Tukey’s post hoc test was used for an analysis with multiple comparisons. A value of * *p* < 0.05 was considered statistically significant. Values of ** *p* < 0.01, *** *p* < 0.001, or **** *p* < 0.0001 were considered highly significant, and *p* > 0.05 (ns) was considered not statistically significant. All analyses were performed using GraphPad Prism 9.0.

## 3. Results

### 3.1. 2’-HC Inhibited the Proliferation of Breast Cancer In Vitro and In Vivo

**Figure 1:**
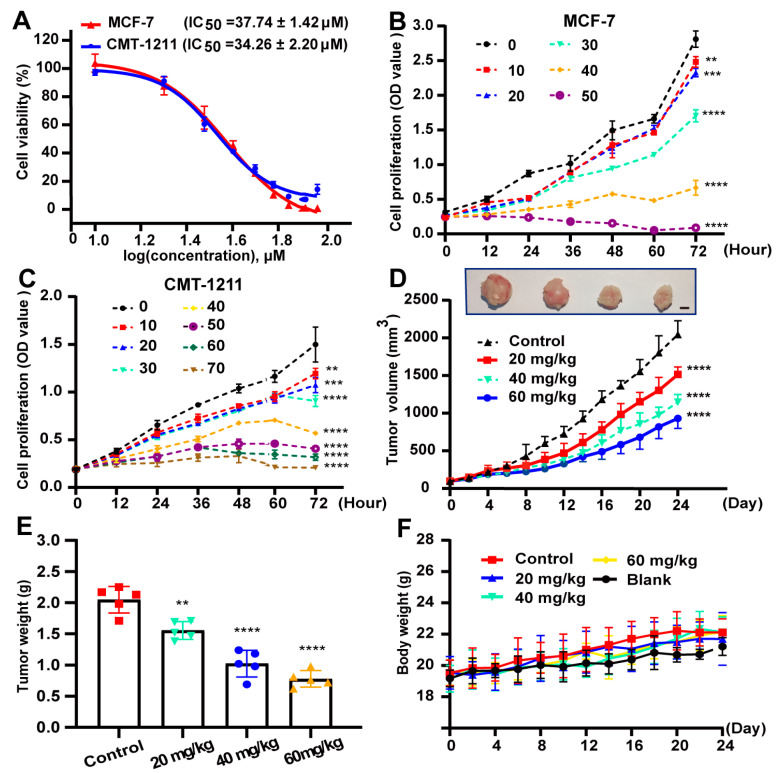
2’-HC inhibited the proliferation of breast cancer in vitro and in vivo. (**A**) MCF-7 and CMT-1211 cells were treated with indicated concentrations of 2’-HC for 24 h. Cell viability was assessed with Cell Counting Kit-8 kits. Data were obtained from three independent experiments (*n* = 3). (**B**,**C**) The proliferation of MCF-7 and CMT-1211 cells treated with different concentrations of 2’-HC was measured with Counting Kit-8 kits. Data were obtained from three independent experiments (*n* = 3). (**D**–**F**) A CMT-1211 xenograft mouse model was established to assess the anticancer effect of 2’-HC in vivo (*n* = 5 per group). Intraperitoneal injection of 2’-HC and measurements of tumor volume (**D**; scale bar, 0.5 cm) and body weight (**F**) of the mice were processed every two days. After 24 days of 2’-HC treatment, all mice were euthanized and the tumor samples were weighed (**E**). Data are presented as the mean ± SD. ** *p* < 0.01, *** *p* < 0.001, **** *p* < 0.0001, ns (not significant; *p* ≥ 0.05).

We first evaluated the effect of 2’-HC on cell viability and cell proliferation. As shown in Figure 1A, 2’-HC suppressed cell viability in a dose-dependent manner, and the IC50 of 2’-HC on the two breast cancer cell lines MCF-7 and CMT-1211 was 37.74 ± 1.42 μM and 34.26 ± 2.20 μM, respectively. In addition, 2’-HC inhibited the proliferation of breast cancer cells, with statistically significant differences observed between the control and treated groups due to 10 μM of 2’-HC treatment on both cell lines (Figure 1A,C). We continued to evaluate whether 2’-HC could exert the anti-proliferation effect of breast cancer in vivo. As shown in Figure 1D,E, 2’-HC management decreased the volume and weight of the tumor in the mouse xenograft tumor models, whereas it had no significant effect on the mice body weight even at a high dose (Figure 1F).

### 3.2. 2’-HC Induced Autophagy and Promoted Autophagy Flux in Breast Cancer Cells

**Figure 2:**
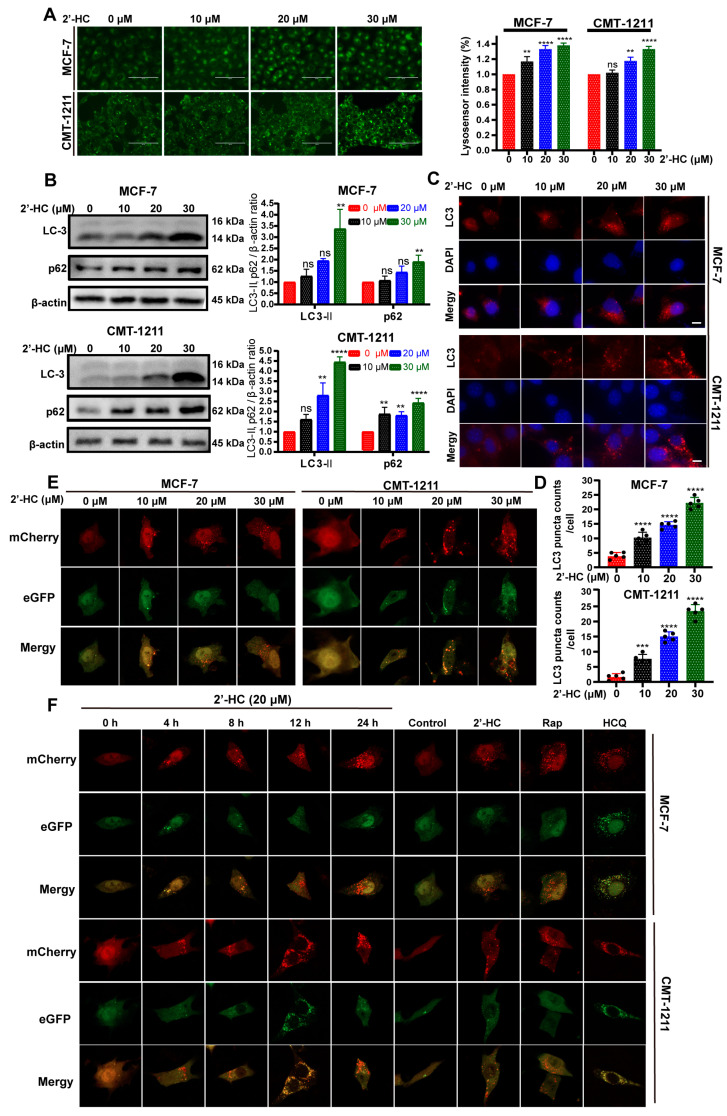
2’-HC induced autophagy and promoted autophagy flux in breast cancer cells. (**A**) The number of acidic vesicular organelles of MCF-7 and CMT-1211 cells was measured with lysosensor reagent after 2’-HC treatment, reflected by green fluorescence intensity within cells. Scale bar, 100 μm. Data were obtained from three independent experiments (*n* = 3). (**B**) Western blot analyses of LC-3 and p62 in cells treated with 2’-HC for 24 h. β-actin was used as an internal control. Data were obtained from three independent experiments (*n* = 3). (**C**,**D**) Immunofluorescence staining of LC3 in MCF-7 and CMT-1211 cells that were treated with 2’-HC for 24 h. Scale bar: 10 μm. Data were obtained from five independent experiments (*n* = 5). (**E**) The MCF-7 and CMT-1211 cells were transfected with a plasmid encoding mCherry-eGFP-LC3 and treated with 2’-HC for 24 h. Images were captured with confocal microscopy. Scale bar: 10 μm. (**F**) The MCF-7 and CMT-1211 cells were transfected with a plasmid encoding mCherry-eGFP-LC3 and treated with 20 μM 2’-HC for 4, 8, 12, 24 h. Treatment with Rapamycin (Rap, 1 μM) and Hydroxychloroquine (HCQ, 20 μM) for 24 h was used as positive and negative controls, respectively. Scale bar: 10 μm. Data are presented as the mean ± SD. ** *p* < 0.01, *** *p* < 0.001, **** *p* < 0.0001, ns (not significant; *p* ≥ 0.05).

The number of acidic vesicular organelles increased after 2’-HC treatment (Figure 2A). This phenomenon implied that autophagy might be involved in 2’-HC-induced cell death as autophagy is characterized by increased acidic vesicular organelles (autophagosomes and autolysosomes). To confirm this hypothesis, we first detected the autophagy marker protein LC3-II expression with a Western blot. The expression of LC3-II increased after 2’-HC treatment (Figure 2B), consistent with the results of the immunofluorescence staining assay, which showed an increased number of LC3-puncta (Figure 2C,D). These results prove that 2’-HC promoted autophagy vesicle accumulation in breast cancer cells.

p62 expression is generally considered an indicator of autophagic flux, as p62 degrades after the fusion of autophagosomes with lysosomes. Thus, an increased level of p62 is correlated with autophagic flux inhibition. In this experiment, the expression of p62 elevated after 2’-HC treatment (Figure 2B), suggesting that there might be an inhibition of autophagic flux. To verify the influence of 2’-HC on autophagic flux, cells were transfected with the mCherry-eGFP-LC3 plasmid. In the early stage of autophagy, both mCherry and eGFP signals are detectable and exhibit a yellow signal after colocalization. However, the eGFP signal is susceptible to acidic environments and is extinguished upon the fusion of autophagosomes with lysosomes, whereas the mCherry signal remains stable under acidic conditions; only the mCherry signal is detectable after autophagosomes fuse with lysosomes. As shown in Figure 2E, after treatment of 2’-HC for 24 h, the number of intracellular red puncta dramatically increased. In contrast, the level of green puncta only exhibited slight elevation, giving rise to more red-only puncta after colocalization. This result suggests that 2’-HC did not inhibit autophagic flux. Instead, it induced continuous autophagic flux in breast cancer cells. To further verify this hypothesis, fluorescence was detected at various time points to observe the dynamic changes in autophagic flux. The number of yellow puncta increased rapidly within 4 h after 2’-HC treatment, followed by quenching of green puncta, and ultimately resulted in abundant cytoplasmic red-only puncta at the time point of 24 h (Figure 2F). Moreover, Rap (autophagy inducer) and HCQ (late-stage autophagy inhibitor) were used as controls in this experiment. The HCQ-treated group showed an accumulation of abundant yellow puncta, which implied an interruption of autophagic flux, while the group treated with Rap exhibited the same result as the group treated with 2’-HC (Figure 2F), suggesting that 2’-HC played a similar role to Rap in the autophagy process. Taken together, 2’-HC induced autophagy and promoted autophagy flux in breast cancer cells.

### 3.3. 2’-HC Induced Autophagy-Dependent Apoptosis of Breast Cancer Cells

**Figure 3:**
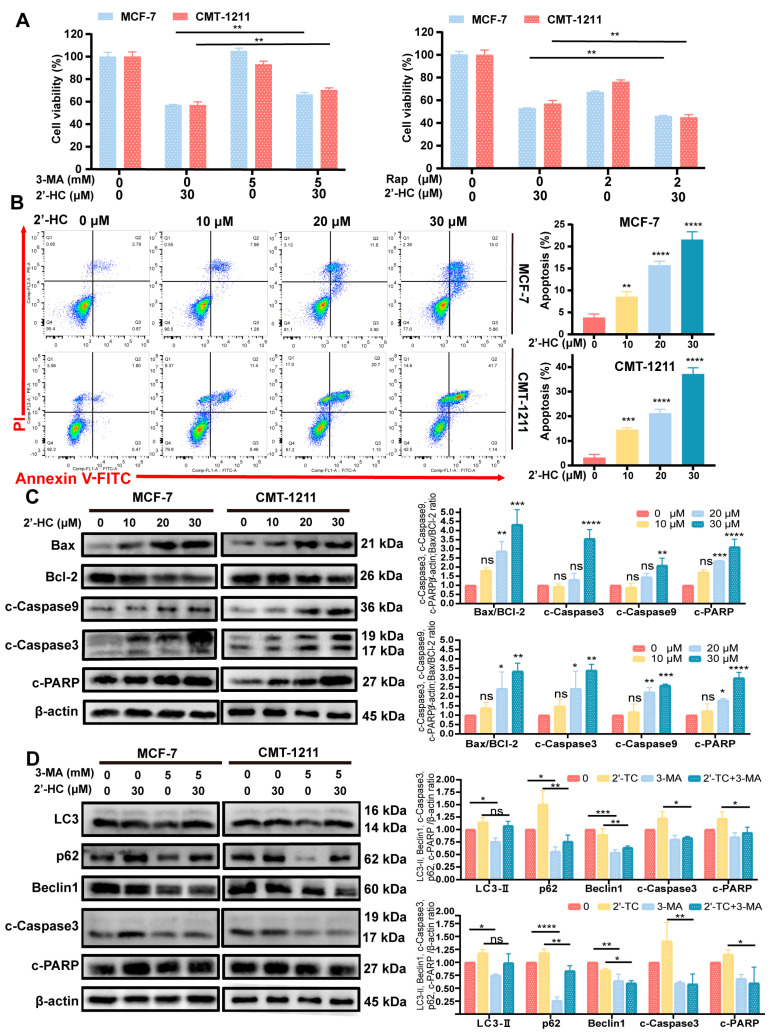
2’-HC induced autophagy-dependent apoptosis of breast cancer cells. (**A**) Cell viability of MCF-7 and CMT-1211 cells was assessed after treatment of 2’-HC (30 μM) or Rapamycin (Rap, 2 μM)/3-Methyladenine (3-MA, 5 mM) or their combination with Cell Counting Kit-8 kits. Cells were pretreated with Rap for 1 h and 3-MA for 2 h before treatment with 2’-HC for 24 h. (**B**) Cell apoptosis assays of MCF-7 and CMT-1211 cells treated with 2’-HC for 24 h using flow cytometer. Cells were collected and labelled with Annexin V-FITC and PI. (**C**) Western blot analyses of Bax, Bcl-2, cleaved caspase-9 (c-Caspase9), cleaved caspase-3 (c-Caspase3), and cleaved-PARP (c-PARP) in cells treated with 2’-HC for 24 h. β-actin was used as an internal control. (**D**) Western blot analyses of LC3, p62, Beclin1, c-Caspase-3, and c-PARP in cells treated with 2’-HC (30 μM) and 3-MA (5 mM) alone or in combination. Cells were pretreated with 3-MA for 2 h before treatment with 2’-HC for 24 h. β-actin was used as an internal control. Data are presented as the mean ± SD of three independent experiments (*n* = 3). * *p* < 0.05, ** *p* < 0.01, *** *p* < 0.001, **** *p* < 0.0001, ns (not significant; *p* ≥ 0.05).

Cell viability was partly reversed after pretreatment with 3-MA (an early-stage autophagy inhibitor) (Figure 3A), suggesting that there were other mechanisms underlying 2’-HC-induced cell death. Apoptosis has been widely reported in multiple types of cancer cells treated with chalcones, and this motivated us to investigate whether apoptosis is associated with 2’-HC-induced cell death. As depicted in Figure 3B, the proportion of apoptotic cells escalated in a dose-dependent manner following 24 h of 2’-HC treatment. The Western blot analysis illustrated that 2’-HC triggered cleavage of caspase-9, caspase-3, and PARP proteins and augmented the expression of pro-apoptotic protein Bax, while suppressing the expression of the anti-apoptotic protein Bcl-2 (Figure 3C). These results indicate that 2’-HC triggered apoptosis in breast cancer cells. Furthermore, to assess whether 2’-HC-induced apoptosis is associated with autophagy, we pretreated cells with 3-MA. Figure 3D shows that 3-MA downregulated the expression of autophagy-related proteins and reversed the increased cleavage of caspase-3 and PARP by 2’-HC. These findings suggest that 2’-HC induced autophagy-dependent apoptosis in breast cancer cells.

### 3.4. Transcriptomic Changes in 2’-HC-Treated Breast Cancer Cells and Network Pharmacological Analysis of 2’-HC

**Figure 4:**
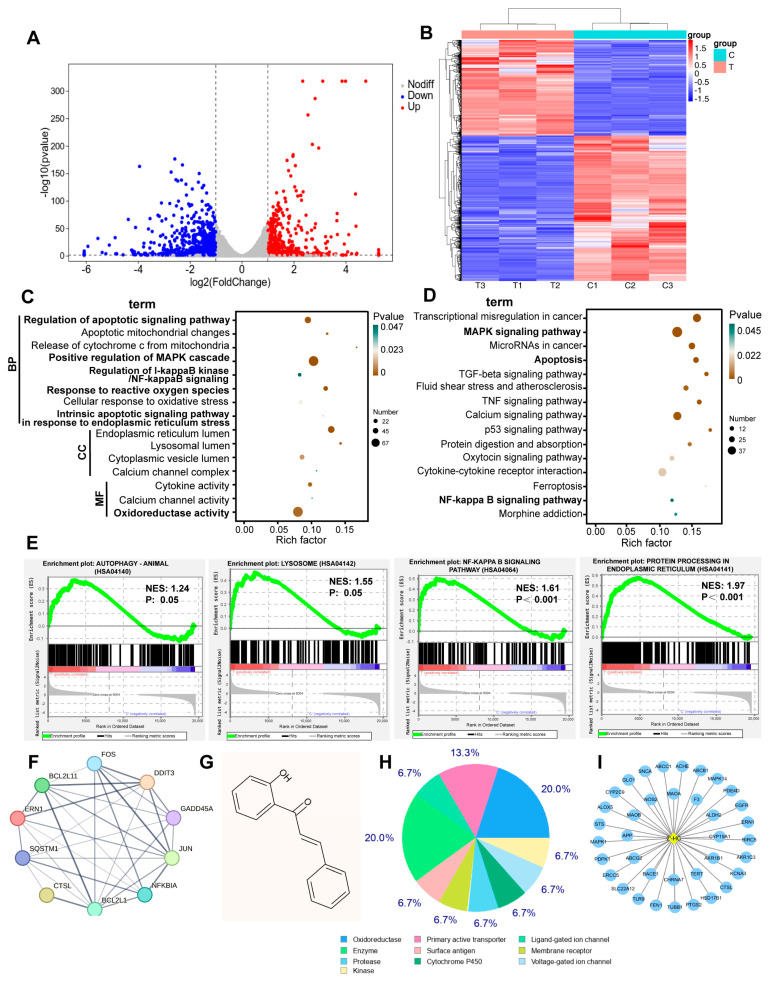
Transcriptomic changes in 2’-HC-treated breast cancer cells and network pharmacological analysis of 2’-HC. (**A**) Volcano plot and (**B**) cluster analysis indicated upregulated and downregulated DEPs of MCF-7 cells. (**C**,**D**) The results of GO and KEGG analysis of all DEPs in 2’-HC-treated group. (**E**) Gene Set Enrichment Analysis (GSEA). NES: normalized enrichment score; p: nominal *p*-value. (**F**) The protein–protein interaction network of hub genes analyzed with STRING. (**G**) The chemical structural formula of 2’-TC attained from PubChem. (**H**,**I**) Results of the potential target genes of 2’-HC predicted with SwissTargetPrediction. MCF-7 cells were treated with 30 μM of 2’-HC for 24 h. There were three duplicate samples in each group (*n* = 3).

To further identify the possible molecular mechanisms underlying the antitumoral activity of 2’-HC, we conducted an RNA sequencing analysis and analyzed the transcriptional changes in MCF-7 cell lines with 2’-HC treatment. There were a total of 1254 DEGs in the 2’-HC-treated group compared with the control group, among which 497 were upregulated and 757 were downregulated (Figure 4A). A cluster analysis shows that expression patterns of DEGs in samples within the control or treated group were similar but exhibited a significant difference in samples between control and treated groups (Figure 4B). The GO database is used to define gene functions, which can be divided into the molecular function (MF), biological process (BP), and cellular component (CC). Significant enrichments of DEGs in the GO analysis included apoptotic signaling pathways, the release of cytochrome c from mitochondria, the positive regulation of the MAPK cascade, the regulation of I-κB/NF-κB signaling, the response to reactive oxygen species, the endoplasmic reticulum lumen, etc. (Figure 4C). The KEGG database was utilized for pathway annotation. In the KEGG pathway analysis, DEGs were significantly enriched in terms of apoptosis, the MAPK signaling pathway, the NF-κB signaling pathway, calcium signaling pathways, etc. (Figure 2D). The GSEA analysis revealed that autophagy, lysosomes, the NF-κB signaling pathway, and protein processing in the endoplasmic reticulum were enriched in the 2’-HC-treated group (Figure 4E). The above enrichment analysis not only verified that autophagy and apoptosis were involved in the effect of 2’-HC against breast cancer cells, which is consistent with our previous results, but also led us to investigate the related pathways regulated in 2’-HC-induced cell death. Figure 4F shows the protein–protein interaction network of hub genes related to apoptosis, autophagy, endoplasmic reticulum stress, and MAPK and NF-κB signaling pathways, implying the multiple biological mechanisms underlying the antitumoral activity of 2’-HC against breast cancer cells, and they are closely linked. 

Furthermore, we predicted the potential target genes of 2’-HC using SwissTargetPrediction. The molecular structure of 2’-HC is shown in Figure 4G. Results show that there were 37 predicted target genes (Figure 4I), and most of them were classified as oxidoreductase (Figure 4H), suggesting that 2’-HC is likely to exert an antitumoral effect by targeting the process of redox reactions.

### 3.5. 2’-HC Regulated MAPK/NF-κB Signaling Pathways and Inhibited Migration/Invasion of Breast Cancer Cells

**Figure 5:**
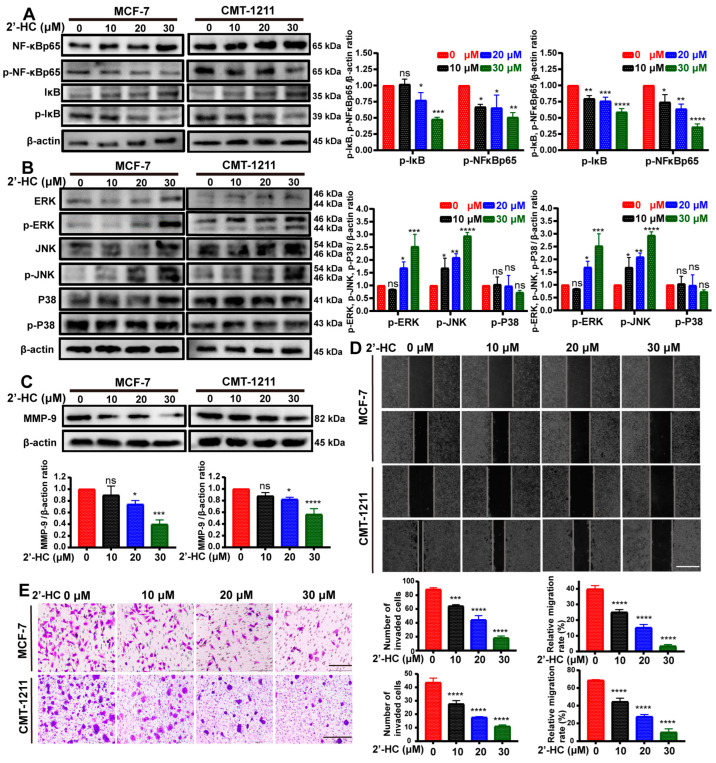
2’-HC regulated MAPK/NF-κB signaling pathways and inhibited migration/invasion of breast cancer cells. (**A**–**C**) Western blot analyses of MCF-7 and CMT-1211 cells treated with 2’-HC for 24 h. β-actin was used as an internal control. p-NF-κBp65 and p-IκB (**A**); p-ERK, p-JNK, and p-P38 (B); MMP-9 (**C**). (**D**) Wound healing assay of MCF-7 and CMT-1211 cells treated with 2’-HC for 24 h to measure the cell migration ability. Scale bar: 100 μm. (**E**) Transwell assay of MCF-7 and CMT-1211 cells treated with 2’-HC for 24 h to measure the cell invasion ability. Scale bar: 200 μm (MCF-7); 500 μm (CMT-1211). Results are presented as the mean ± SD of three independent experiments (*n* = 3). * *p* < 0.05, ** *p* < 0.01, *** *p* < 0.001, **** *p* < 0.0001, ns (not significant; *p* ≥ 0.05).

Subsequently, we investigated whether NF-κB and MAPK signaling pathways were involved in regulating 2’-HC in breast cancer cells through a Western blot analysis. As shown in Figure 5A, the expression of p-IκB and p-NF-κBp65 was significantly reduced after 2’-HC treatment, implying an inhibition of the NF-κB pathway. Meanwhile, levels of p-ERK and p-JNK elevated in the 2’-HC-treated group whereas the expression of p-p38 remained unaltered (Figure 5B), suggesting that extracellular regulated protein kinases (ERKs)/mitogen-activated protein kinase (MAPK) and c-Jun N-terminal kinase (JNK)/MAPK but not p38/MAPK participated in the regulation of 2’-HC on breast cancer cells.

Matrix metalloproteinase-9 (MMP9) is a downstream protein of the NF-κB pathway known to regulate cell migration and invasion. Since 2’-HC could suppress the activation of the NF-κB pathway, we wondered if it could inhibit the migration and invasion of breast cancer cells. To verify this hypothesis, we first detected the expression of MMP9. As expected, MMP9 was downregulated after 2’-HC treatment (Figure 5C). The wound healing assay and transwell assay revealed that the migration rate and the number of invaded cells decreased in a dose-dependent manner in the 2’-HC-treated group (Figure 5D,E). These results imply that 2’-HC could inhibit the migration and invasion of breast cancer cells.

### 3.6. 2’-HC Increased the Intracellular ROS Levels and Triggered ERS of Breast Cancer Cells

**Figure 6:**
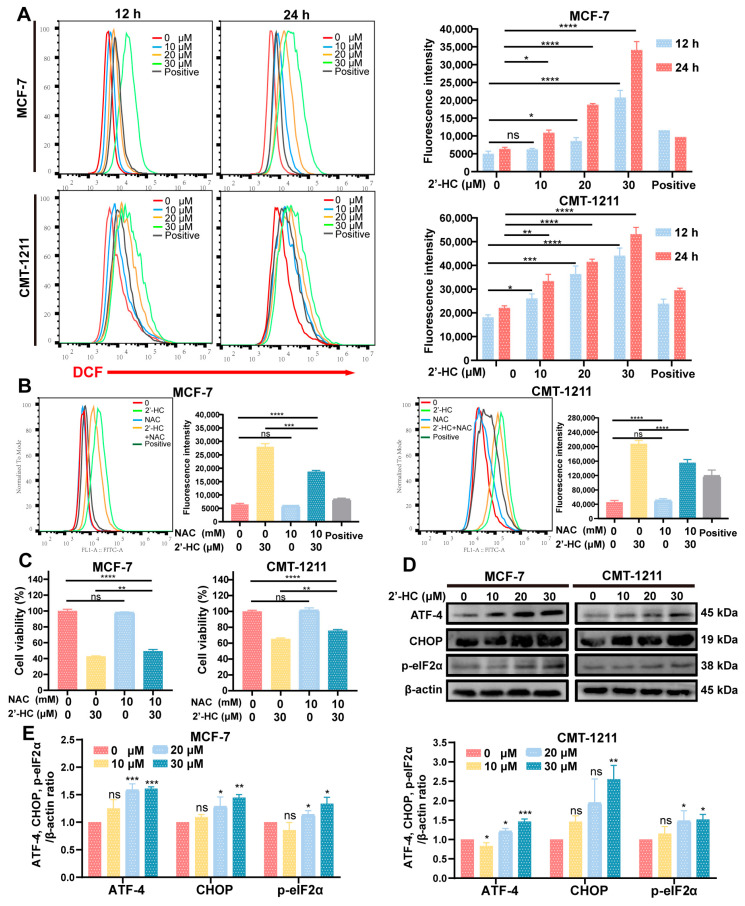
2’-HC increased the intracellular ROS levels and triggered ERS of breast cancer cells. (**A**) Flow cytometry was used to detect the level of ROS in MCF-7 and CMT-1211 after treatment with different concentrations of 2’-HC for 12 h or 24 h. Rosup was used in the ROS-positive control group at a concentration of 50 μL/mL for 20 min of stimulation. (**B**) The level of ROS in MCF-7 and CMT-1211 after treatment with 2’-HC and N-acetylcysteine (NAC) alone or in combination. Cells were pretreated with 10 mM NAC for 2 h before being treated with 2’-HC for another 24 h. (**C**) Cell viability was assessed in MCF-7 and CMT-1211 cells after treatment with 2’-HC and NAC alone or in combination. Cells were pretreated with 10 mM NAC for 2 h before being treated with 30 μM 2’-HC for another 24 h. (**D**,**E**) Western blot analyses of ATF-4, CHOP, and p-eIF2α in cells treated with 2’-HC for 24 h. β-actin was used as an internal control. Data are shown as the mean ± SD of three independent experiments (*n* = 3). * *p* < 0.05, ** *p* < 0.01, *** *p* < 0.001, **** *p* < 0.0001, ns (not significant; *p* ≥ 0.05).

In normal cells, the intracellular level of ROS is relatively low, which is fundamental for maintaining essential physiologic functions of cells. However, an excessive accumulation of ROS causes cellular injury and may induce autophagy and apoptosis in cells. According to the results of the GO enrichment analysis and the prediction of 2’-HC target genes, it was found that responses to reactive oxygen species and genes related to redox reactions were highly engaged in 2’-HC treatment (Figure 4C). This finding encouraged us to examine the level of ROS after 2’-HC treatment. As shown in Figure 6A, intracellular ROS increased dramatically in both dose- and time-dependent manners after exposure to 2’-HC. To investigate whether the increased ROS level was responsible for 2’-HC-induced cell death, cells were pretreated with a ROS scavenger (NAC). Results show that NAC decreased the cells’ intracellular ROS level (Figure 6B). However, the cell death caused by 2’-HC was only slightly reversed by NAC (Figure 6C). These results imply that the increased accumulation of intracellular ROS played a minor role in 2’-HC-induced cell death.

ERS can be triggered by an excessive generation of ROS and causes autophagy and apoptosis in cells. Moreover, GO and GSEA analyses show that responses to endoplasmic reticulum stress and protein processing in the endoplasmic reticulum were significantly enriched in the 2’-HC-treated group (Figure 4C,E). Thus, the expression levels of ATF-4, CHOP, and p-eIF2α, indicators of ERS, were measured with a Western blot. Figure 6D,E show that all the proteins were upregulated by 2’-HC, verifying that 2’-HC triggered ERS in breast cancer cells.

### 3.7. In Vivo Regulation of 2’-HC in Breast Cancer

**Figure 7:**
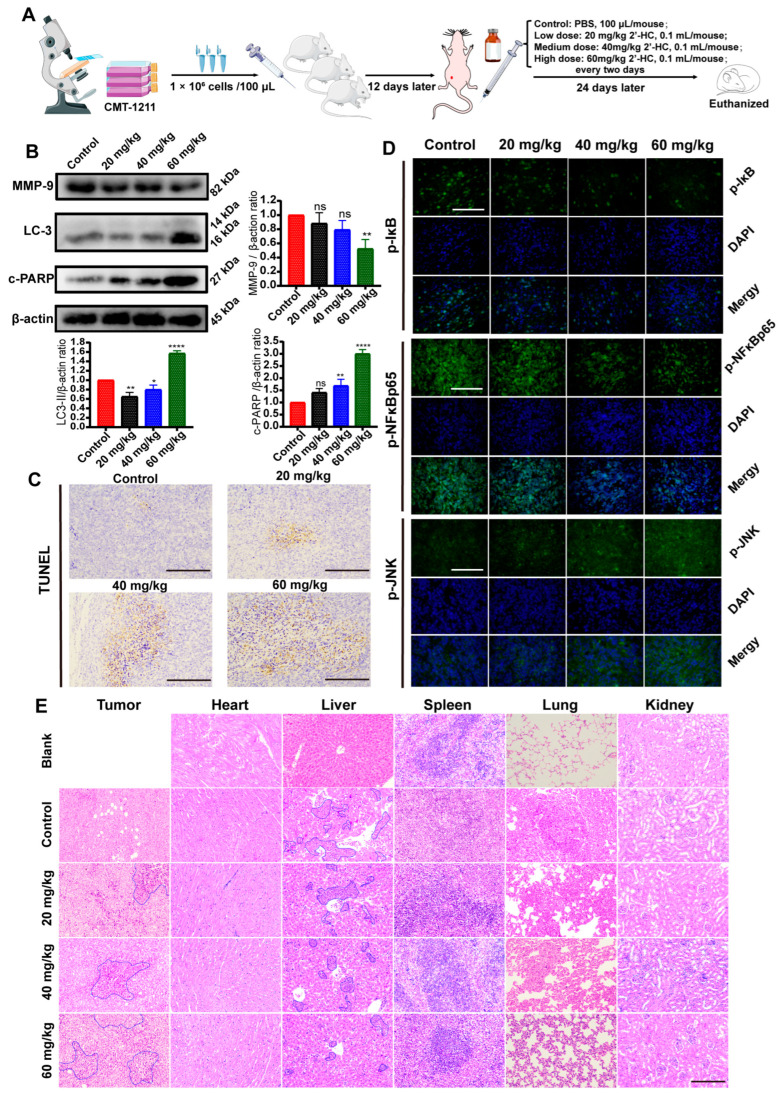
In vivo regulation of 2’-HC in breast cancer. (**A**) Schematic drawing of the procedure of establishing the xenograft mouse model and drug management. (**B**) Western blot analyses of MMP-9, LC-3, and c-PARP in tumor tissues. β-actin was used as an internal control. (**C**) Results of TUNEL assay of tumor tissues. Scale bar: 200 μm. (**D**) Immunofluorescence staining of p-NF-κBp65, p-IκB, and p-JNK of tumor tissues. Scale bar: 200 μM. (**E**) Representative panels of H&E staining of tumor and essential organ (heart, liver, spleen, lung, and kidneys) tissues of mice. The area of necrosis in tumor tissues is marked by blue borders. Blue borders in the liver tissues indicate metastatic cells. Scale bar: 200 μm. All results are shown as the mean ± SD of three independent experiments (*n* = 3). * *p* < 0.05, ** *p* < 0.01, **** *p* < 0.0001, ns (not significant; *p* ≥ 0.05).

To investigate the antitumoral activity of 2’-HC in vivo, a subcutaneous xenograft mouse model was established using CMT-1211 (Figure 7A). Figure 7C shows that the number of TUNEL-positive cells increased in tumor tissues after 2’-HC management, consistent with the results of an elevated expression of c-PARP in the 2’-HC-treated group (Figure 7B). These results suggest that 2’-HC induced apoptosis in xenograft tumors. We also observed an upregulation of LC-3II (Figure 7B) and p-JNK and a downregulation of p-IκB and p-NF-κBp65 following 2’-HC treatment (Figure 7D). These results align with the results of the in vitro experiment, which show the enhancement of autophagy and the JNK/MAPK pathway and the inhibition of the NF-κB pathway.

H&E staining shows that in the control group, there were apparent tumor metastases in the lung and liver (blue border). Meanwhile, in the high-dose treatment group, the range of tumor metastases in the lung and liver significantly reduced (Figure 7E). The Western blot assay exhibits a similar result: 2’-HC decreased metastases in the xenograft mouse model by showing a downregulated MMP9 expression (Figure 7B). Additionally, an extensive range of necrosis, characterized by the pyknosis, fragmentation, and dissolution of the nucleus (blue border), was observed in tumor tissues in the medium- and high-dose groups (Figure 7E). No significant toxic effect on cells or tissues of the critical organs was observed after 2’-HC management (Figure 7E). These results suggest that 2’-HC could exhibit an antitumor effect in vivo without causing significant toxicity to essential organs.

## 4. Discussion

Chalcones are dietary compounds reported to demonstrate the most potent antitumoral effects among the diverse subclasses of flavonoids [8,24], and they naturally occur in hydroxylated forms in most cases [10]. (E)2’-hydroxychalcone (2’-HC) is believed to be the simplest hydroxychalcone and exists in a more thermodynamically stable form. Previous investigations have proved that 2’-HC could significantly suppress the proliferation of cervical, lung, and colon cancer cells [13,14,15]. However, whether it could elicit inhibitory effects on breast cancer cells MCF-7 and CMT-1211 has never been reported before and, thus, remains to be explored. CMT-1211 is a canine-derived cell line. The similarities in epidemiological, tumor morphological, and clinical pathological characteristics between canine and human mammary tumors make the canine mammary tumor model one of the most ideal models for studying human breast cancer [25]. This study, for the first time, demonstrated that 2’-HC could inhibit breast cancer cell proliferation, migration, and invasion in vitro, as well as suppress tumor growth and metastasis in vivo. Mechanistic studies showed that the NF-κB pathway was significantly inhibited by 2’-HC treatment accompanied by an excessive intracellular accumulation of ROS, induction of ERS, and activation of JNK/MAPK. In addition, autophagy-dependent apoptosis was also found in the 2’-HC-induced cell death.

Autophagy is a conserved catabolic process that degrades damaged cytoplasmic proteins and organelles via lysosomes [26]. Cancer cells exhibit enhanced autophagic levels due to their rapid growth and increased metabolism [27], making the modulation of the autophagic process a vital technique to develop effective and low-toxicity anticancer drugs. Our study found that 2’-HC could enhance the initiation of autophagy in breast cancer cells accompanied by a complete autophagic flux. In addition, numerous studies show that the accumulation of intracellular ROS is responsible for overloaded misfolded proteins, which lead to ERS and finally trigger autophagy [28,29]. In the present study, over-productions of ROS and ERS were observed after 2’-HC treatment, implying the potential causes of cellular autophagy. The role of autophagy in tumorigenesis is complex and context-dependent: it can function as a defender of cellular homeostasis as well as an inducer of cancer cell death [30,31,32]. To identify the role of autophagy in regulating 2’-HC on the two breast cancer cell lines, Rap, an inducer of autophagy, and 3-MA, an early-stage autophagy inhibitor, were used in this study. 3-MA could partly reverse the decreasing cell viability caused by 2’-HC, whereas Rap synergistically enhanced the cytotoxicity of the drug on cells. These results imply that 2’-HC-induced autophagy was not cytoprotective but induced autophagic cell death in breast cancer cells.

p62 is a multifunctional protein with multiple domains. In autophagy, p62 serves as an autophagic cargo protein and is degraded after autophagosomes fuse with lysosomes [33]. The accumulation of p62 was typically used as an indicator of a blocked autophagic flux. However, in some situations, p62 serves as a stress response protein and can be aberrantly activated through oxidative stress, drug stimulation, and other stress conditions [34,35]. It tends to increase both at protein and transcript levels. In the present study, p62 was upregulated both at the protein level and transcript level, which could be attributed to the stress conditions induced by 2’-HC stimulation.

Apoptosis is the most common mechanism in chalcone-induced cell death in diverse cancers. This study proved that 2’-HC caused apoptosis in breast cancer cell lines and xenograft mouse tumors. The inhibition of autophagy reduced 2’-HC-induced apoptosis, implying an occurrence of autophagy-dependent apoptosis. Moreover, upon treatment with 2’-HC, members of the MAPK family such as JNK and ERK were activated. Research indicates that JNK plays a crucial role in the cellular stress response, and its activation consequently triggers apoptosis [36]. ERK activation is commonly associated with pro-survival and pro-proliferation processes [37], but there are also substantial studies reporting the involvement of ERK in the regulation of apoptosis [38,39,40]. Elevated ERK levels have been shown to induce apoptosis in cells. These results are consistent with the findings of the present study.

The NF-κB signaling pathway is a pro-survival pathway and has been reported to be upregulated in a majority of cancers, including breast cancer, contributing to the proliferation and metastasis of cancer cells and protecting them from adverse conditions [41]. Targeting this pathway with natural compounds is a promising strategy to inhibit cancer development. A large spectrum of chalcones was identified and studied for their potential to inhibit the NF-κB pathway [42,43,44]. In this study, we verified that 2’-HC significantly inhibited the NF-κB pathway. Additionally, its target genes, Bcl-2 and MMP9, were downregulated accordingly. Therefore, we hypothesize that suppressing the NF-κB pathway contributes to the pro-apoptotic effects and the inhibition of cell migration and invasion by 2’-HC in breast cancer cells. Furthermore, NF-κB is an essential pathway for scavenging intracellular ROS through the regulation of antioxidant enzymes such as superoxide dismutase (SOD2) and ferritin heavy chain (FHC) [45,46]. Thus, the pharmacological inhibition of the NF-κB pathway may cause ROS accumulation and subsequent ERS, resulting in autophagy and apoptosis. This provides a possible explanation for the accumulation of ROS within cells after 2’-HC treatment. Reports are indicating that NF-κB exerts an anti-apoptotic effect through the inactivation of JNK [47]. In this study, both the upregulation of p-JNK and the downregulation of p-p65 were observed after 2’-HC administration. These findings may also be potential reasons for drug-induced apoptosis. Interestingly, the drug-target prediction analysis shows that the predicted target genes of 2’-HC were predominantly classified in oxidoreductases. Since the NF-κB pathway also plays a significant role in regulating redox reactions [48], it is hypothesized that the inhibition of the NF-κB pathway serves as a central hub in the regulation of 2’-HC against breast cancer, which could contribute to the excessive accumulation of ROS, the activation of JNK, and the inhibition of MMP9 and anti-apoptotic protein Bcl-2, and eventually causes autophagy and apoptosis. The significant enrichment of the NF-κB signaling pathway in KEGG, GO, and GSEA analyses further supported this hypothesis. However, this study did not validate the specific mechanism by which 2’-HC inhibits the NF-κB pathway or the precise steps in the NF-κB pathway affected by 2’-HC. Therefore, further research is needed to accomplish it.

## 5. Conclusions

Our study, for the first time, verified that 2’-HC could inhibit breast cancer cell proliferation, migration, and invasion in vitro and suppress tumor growth and metastasis in vivo. The underlying mechanisms focus on the inhibition of the NF-κB pro-survival pathway, which could contribute to the excessive accumulation of cellular ROS, ERS, and autophagy-dependent apoptosis in breast cancer cells. These findings offer new insights into the molecular mechanisms of 2’-HC in combating breast cancer, indicating a promising potential for 2’-HC in future anticancer drug development.

## Data Availability

All the data used and analyzed for this study are available from the corresponding author upon reasonable request. The RNA sequencing data are available in SRA database with an accession number: SRX23210086.

## Abbreviations

2’-HC	(E)2’-hydroxychalone
ROS	reactive oxygen species
ERS	endoplasmic reticulum stress
HCQ	hydroxychloroquine
3-MA	3-methyladenine
NAC	N-acetylcysteine
Rap	rapamycin
DEGs	differentially expressed genes
GSEA	gene set enrichment analysis
KEGG	Kyoto Encyclopedia of Genes and Genomes
GO	gene ontology
STRING	Search Tool for the Retrieval of Interacting Genes
PI	propidium iodide
FITC	fluorescein isothiocyanate
TUNEL	terminal deoxynucleotidyl transferase dUTP nick end labeling
H&E	hematoxylin and eosin
